# Exploration for the Salinity Tolerance-Related Genes from Xero-Halophyte *Atriplex canescens* Exploiting Yeast Functional Screening System

**DOI:** 10.3390/ijms18112444

**Published:** 2017-11-17

**Authors:** Gang Yu, Jingtao Li, Xinhua Sun, Yanzhi Liu, Xueliang Wang, Hao Zhang, Hongyu Pan

**Affiliations:** 1 College of Plant Sciences, Jilin University, Changchun 130062, China; chrisyu_gang@hotmail.com (G.Y.); lijingtao789@126.com (J.L.); xinhua-sun@hotmail.com (X.S.); liuyz8209@mails.jlu.edu.cn (Y.L.); mewxl@163.com (X.W.); 2 College of Resource and Environment, Jilin Agricultural University, Changchun 130062, China

**Keywords:** *Atriplex canescens*, salinity tolerance, yeast expression, halophyte, Gene Othology analysis

## Abstract

Plant productivity is limited by salinity stress, both in natural and agricultural systems. Identification of salt stress-related genes from halophyte can provide insights into mechanisms of salt stress tolerance in plants. *Atriplex canescens* is a xero-halophyte that exhibits optimum growth in the presence of 400 mM NaCl. A cDNA library derived from highly salt-treated *A. canescens* plants was constructed based on a yeast expression system. A total of 53 transgenic yeast clones expressing enhanced salt tolerance were selected from 10^5^ transformants. Their plasmids were sequenced and the gene characteristics were annotated using a BLASTX search. Retransformation of yeast cells with the selected plasmids conferred salt tolerance to the resulting transformants. The expression patterns of 28 of these stress-related genes were further investigated in *A. canescens* leaves by quantitative reverse transcription-PCR. In this study, we provided a rapid and robust assay system for large-scale screening of genes for varied abiotic stress tolerance with high efficiency in *A. canescens*.

## 1. Introduction

The world’s population is on track to grow from 7 to 9 billion in the next 50 years [1]. Securing the reliable production of staple crops to feed these people is the primary task of governments world-wide and is one of the most important concerns for state security. Abiotic stresses (such as drought, salinity and extreme temperature) negatively impact crop growth and productivity. High soil-salt concentrations not only impair the ability of plants to take up enough water for growth and development, but large amounts of Na^+^ and Cl^−^ uptake negatively impacts growth by impairing metabolic processes and decreasing photosynthetic efficiency [2]. In the face of salt stress, plants use coordinated strategies to combat salt stress which associates with Reactive oxygen species (ROS) generation and detoxification pathways, signal transduction, osmo-regulation or ion homeostasis by osmoprotectants, and regulated expression of salt responsive genes and transcription factors [3]. Exogenous application of chemicals including compatible solutes and hormones, like glycine betaine, glutathione, polyamines, nitric oxide (NO), brassinosteroids (BRs) and salicylic acid (SA), were also reported as an effective approach to increase the salt resistance in certain plant species [4].

The identification of elements of salt stress resistance is still a major topic in plant breeding and genetic engineering. Currently, plant abiotic stress research is mainly focused on herbaceous plants, while abiotic stress resistance determinants in woody halophytes are as yet rarely investigated. Notably, *Casuarina glauca*, an actinorhizal plant with N2-fixing bacteria of the genus *Frankia*, is a good model to investigate relationship among plant, abiotic stress and symbiotic microbes and its salt-resistant characteristics have been extensively characterized at the physiological level, such as photosynthesis, nitrogen metabolism, membrane integrity and antioxidative system and metabolome [5,6,7,8]. Halophytes have been considered to be an elite resistance gene resource, and research has become focused on isolation and functional characterization of their genes [9,10]. Whole genome sequence information of laboratory model plants including *Arabidopsis thaliana*, and *Thellungiella salsuginea* has made possible comparative analysis of a glycophyte (*A. thaliana*) and a haplophyte (*T. salsuginea*) [11]. Such comparative genomics and experimental analyses have demonstrated that gene families categorized to cation transport, abscisic acid signaling, and wax production in *T. salsuginea* might contribute to its success in stressful environments [11]. Another study focusing on physiological and proteomic analyses of salt stress responsive genes in the halophyte *Halogeton glomeratus* proposed that photosynthesis, energy production, ion homeostasis and oxygen radical scavenging enzymes are involved in maintaining homeostasis under conditions of salt stress [9]. Transcriptomics and subsequent microarray analysis identified genes involving in osmotic and ionic homeostasis, redox equilibrium and signal transduction during salt treatment of another halophyte *Atriplex centralasiatica* [12]. Advances in genome sequencing is driving a revolution in genomics, transcriptomics and proteomics analysis in the investigation of molecular and biochemical mechanisms that underpin biological functions and is providing strategies for crop improvement [13]. But the raw data provided by the “omics” approaches does not identify precise controlling factors in many of these biological processes.

The elite halophyte *Atriplex canescens* (four-wing saltbush), a member of the *Chenopodiaceae*, is indigenous in arid and semi-arid areas of western North America. *A. canescens* exhibits tolerance to salinity, drought, heavy metals and low temperature and the plant has been employed in phytoremediation of saline–alkali and heavy-metal contaminated soils [14]. Therefore, the plant is a source of genes that could be employed in the genetic manipulation of crops for improvements in salt, drought and low temperature stress [14]. Yeast expression systems have been extensively used for protein functional characterization and protein production, combining advantages in rapid growth and facile genetic manipulation with the relevance of a eukaryotic expression system, such as capacity for post-translational modifications [15]. Some recent studies demonstrated that the yeast expression system is suitable to isolate genes responsible for salt-, drought-, and high temperature-resistance in *Jatropha curcas*, tomatos and *Salicornia europaea*, respectively [16,17,18,19]. Since little is known about molecular determinants and mechanisms for adaption to salt stress in woody halophyte *A. canescens*, we describe a yeast expression and screening system to rapidly and efficiently isolate salt-responsive genes from that source.

## 2. Results

### 2.1. Generation of the Yeast Expression cDNA Library

**Figure 1:**
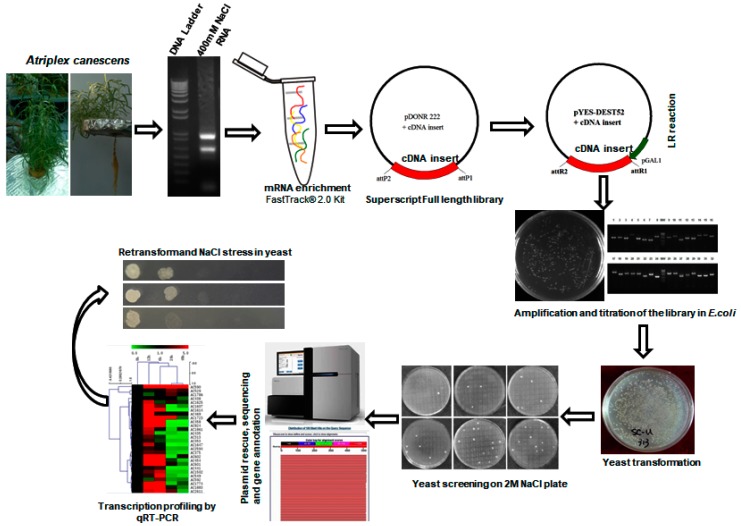
Schematic illustration of cDNA library construction and yeast functional screening. Generally, 400 mM NaCl treated *A. canescens* was subjected to RNA isolation and then cDNA library construction. The cDNA library pool was transformed into yeast cells and subsequently treated with salt under induction conditions. Finally, the survived yeast was turned to plasmid preparation and the plasmids were sent for sequencing and bioinformatics analyses.

A yeast expression library was constructed using the SuperScript full-length Library Construction Kit II, following manufacturer’s instructions (Figure 1). Approximately, 1.76 × 10^6^ colony-forming units (CFU) of *E. coli* transformants harboring *A. canescens* cDNA inserts by BP reaction were generated in pDONR222 with a recombinant rate of 91%. And the primary cDNA library was transferred to a yeast expressional destination vector pYES-DEST52 by LR reaction with a recombinant rate about 95% [20]. The size of cDNA inserts ranged from 0.6 to 2 kb and the average inserted fragment is sized above 1 kb (Table 1).

### 2.2. Identification of Salt Tolerance-Related Genes from A. canescens

**Figure 2:**
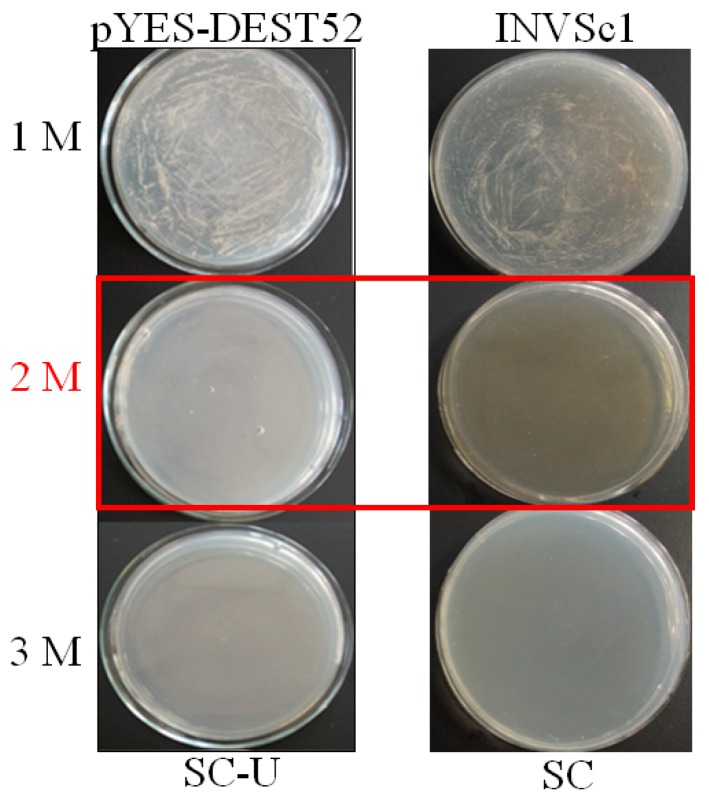
Optimization of NaCl concentrations for yeast functional screening. Yeast cells INVSc1 harboring pYES-DEST and control were induced with 2% galactose and streaked on Synthetic Complete without Uracil (SC-U) and SC medium, respectively. The plates were kept at 28 °C for 72 h and photographed. The concentration (2M NaCl highlight in red) without emerging yeast colonies were used for screening.

**Figure 3:**
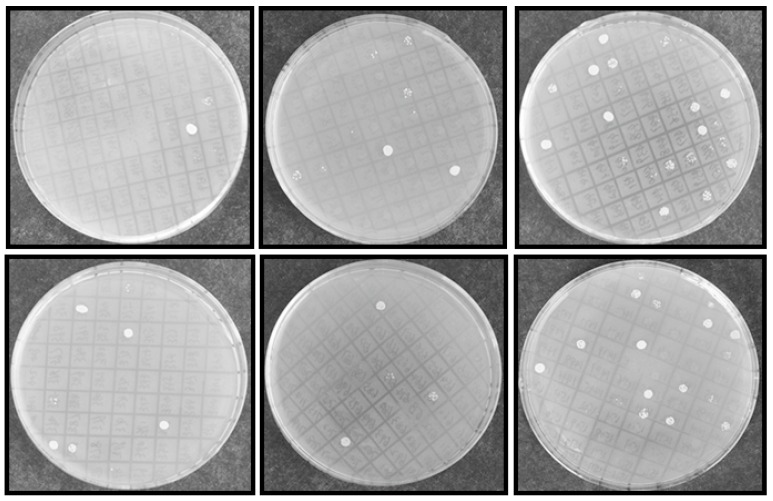
Yeast transformants exhibited NaCl resistance on SC-U plates. INVSc1 competent cells were transformed with library plasmid DNA and streaked on 2% glucose SC-U plate and incubated for 72 h at 28 °C. After yeast colony was visualized, more than 1 × 10^5^ transformants were picked up randomly and induced with 2% galactose for 24 h, 2 μL transformed yeast was spotted on SC-U solid plates (2% agar + 2% galactose) with 2 M NaCl and then incubate at 28 °C for 72 h and photographed.

**Figure 4:**
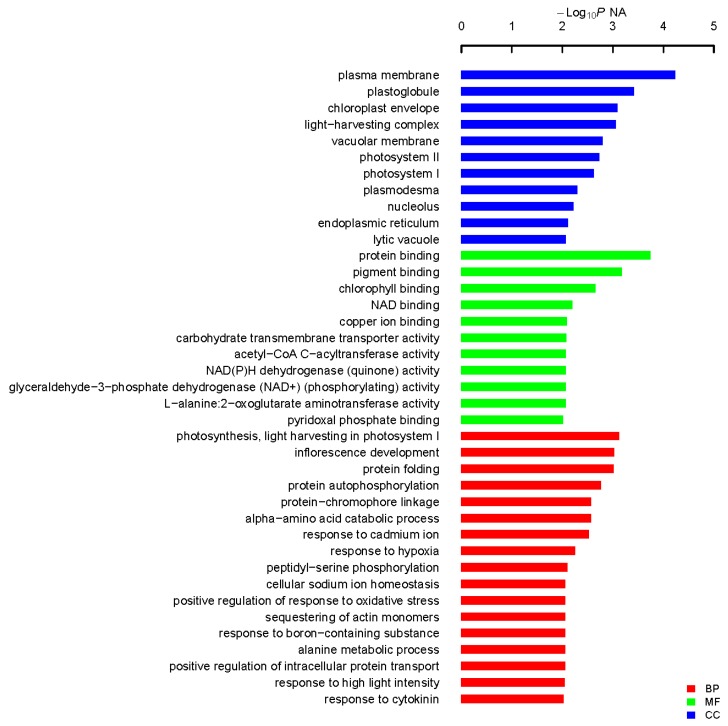
Functional categorization of isolated genes in *A. canescens* seedlings exposed to salinity. Fisher’s exact test was used and *p* < 0.05 was considered to indicate a statistically significant difference to identify biological process (BP), cellular component (CC) and molecular function (MF).

From initial screening, 2 M NaCl was chosen to use as a stringent selection concentration (Figure 2). As shown in Figure 3, 53 tolerant colonies were recovered (Figure 3) from approximately 1 × 10^5^ yeast transformants on the Synthetic Complete without Uracil (SC-U) medium containing 2 M NaCl, and most of the yeast were not able to survive on 2 M NaCl plates. Plasmids isolated from all 53 salt-tolerant yeast transformants were back-transformed into *E. coli* for propagation and sequencing. Insert sequences from all 53 yeast colonies were identified by BLAST analysis and the sequence information were deposited in GenBank. Corresponding GenBank accession numbers are listed in Table 2. Thirty-four out of these 53 cDNA sequence contain the full ORF (open reading frame) (Table 2). All of these genes are homologous to the known genes in the other plants. Gene ontology (GO) classification of the isolated genes was performed to identify the functional processes. Overall, these isolated genes were mainly membrane associated and with binding and catalytic activity categories (Figure 4).

### 2.3. Expression Profiles of Selected Salt Resistance Genes under Abiotic Stress

**Figure 5:**
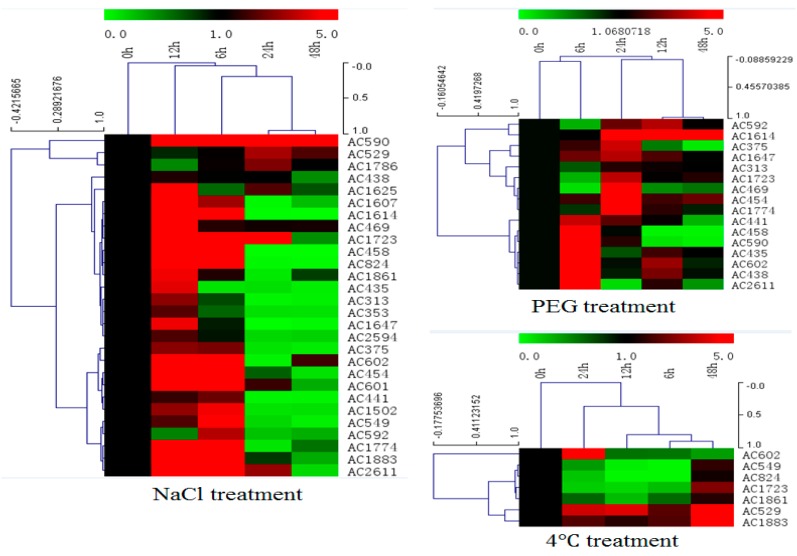
Gene expression profiles of 28 isolated genes in *A. canescens* under various stressors by quantitative real-time RT-PCR analysis. Hierarchical clustering was built using MeV software. Each gene is demonstrated by a single row of colored boxes, and a single column represents different time points with stress treatment. The scale representing the relative signal intensity values is shown above. Induction (or repression) ranges from pale to saturated red (or green) with a fold change scale bar (in log 2) shown up the clusters.

In order to investigate expression profiles of isolated salt resistance genes under salt treatment, total RNA was isolated from leaf tissues of *A. canescens* that had been treated with 400 mM NaCl for various time points (0, 6, 12, 24 and 48 h) and subjected to cDNA synthesis and real-time PCR (Figure 5 and Figure S1 in the Supplementary Material). From the qRT-PCR data, most of the selected genes (28 genes) could be induced by salt treatment at an early time point (6 h, 12 h), transcript accumulation at highest level was 12 h after treatment, and transcript signals decreased from 24 to 48 h after treatment. Transcript accumulation increase varied from 1- to 70-fold for different genes. Furthermore, the expression patterns under drought and cold treatment for these selected genes were investigated by qRT-PCR as well. Two-thirds of the genes (16/28) induced by salt could be induced by drought treatment as well (Figure 5 and Figure S2 in the Supplementary Material). Only seven genes induced by salt could be induced by low temperature treatment (Figure 5 and Figure S3 in the Supplementary Material). Only one gene among them, encoding a late embryogenesis abundant protein, could be induced by all these three treatments simultaneously (Figure S4 in the Supplementary Material).

### 2.4. Recapturing Salt Resistance Assay of Isolated Genes

**Figure 6:**
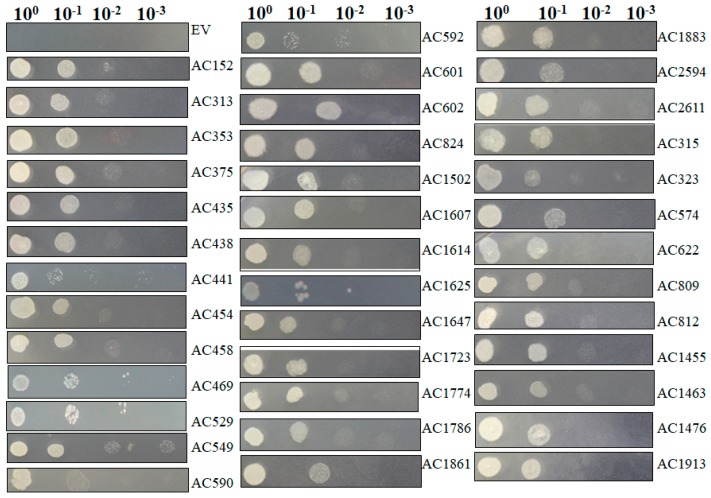
Functional analyses of selected salt resistance related genes in transformed yeast cells. Salt resistance related genes screened from primary screening in large scale selection were retransformed into yeast cells. Then the induced yeast was spotted with a series of dilutions on SC-U plate plus 2 M NaCl. Photographs were taken 72 hpi.

The isolated plasmids were retransformed into new yeast cells individually and salt resistance capacity was retested by spotting those induced yeast cells on SC-U medium containing 2 M NaCl (Figure 6) under conditions in which yeast cells containing pYES-DEST52 (control) were not able to survive. Most of tested pYES2-*Acgenes* (39/53, 73.6%) retained the ability to survive on SC-U medium with salt (Table 2). The genes that exhibited upregulated expression profiles in Figure 5 showed the capacity to survive on the salt-stressed SC-U medium. Under these conditions, the retransformed yeast cells survived 2 M NaCl stress after 1:10 dilution, but rarely at further diluted concentrations, which might be because of the stringent NaCl concentrations. 

## 3. Discussion

Salinity stress limits plant growth and development, leading to productivity loss. An understanding of plant abiotic stress responses, including determinants of salt stress tolerance, would be of importance for both basic and applied plant science research [21]. The woody halophyte Atriplex species, including *A. canescens*, are well-known bushes for their tolerance to salinity, drought, heavy metals and extreme temperature and are considered to serve as a potential candidate for the salt-responsive genes and promoters [3,14]. A few reports described abiotic stress related genes isolated from *A. canescens.* We characterized a heavy metal-resistant protein AcHMA1 from *A. canescens* and transgenic yeast cells harboring AcHMA1 showed a significantly improved survival rates on medium supplemented with iron, salt, alkaline, osmotic and oxidant stress [22]. Another two reports from our lab identified AcNIP5;1 and AcPIP2, two isoforms of aquaporin from *A. canescens*, which altered the resistance phenotypes in transgenic *A. thaliana* plants in the face of either drought or salt stress [23,24]. More recently, an ErbB3-binding protein, AcEBP1 from *A. canescens*, was demonstrated to function as a negative regulator responding to salt and osmotic stress [25].

In order to further explore molecular determinants of salt tolerance in *A. canescens*, a gateway cloning system compatible cDNA library was generated from *A. canescens* plants exposed to 400 mM NaCl [20]. 400 mM NaCl is high concentration for plants, but it does not affect *A. canescens’* normal growth, which could significantly enhance *A. canescens’* leaf osmotic potential compared to the control plants with no salt treatment in a recent report [26]. Lasting salt treatment, such as 48-h exogenous application, could allow us to isolate abundant and important salt tolerance determinants. In the present study, we cloned the cDNA library into a yeast expression system, followed by yeast *S. cerevisiae* INVSc1 transformation. Yeast expression system was proved to be a rapid and robust assay system for large-scale screening of genes for abiotic stress tolerance with attractive features like rapid growth in culture, eukaryotic expression and conserved signaling and regulatory pathways, and post-translational modification process with the advantage of facile genetic manipulation [16,17,18,27]. Heterologous gene expression taking use of the conserved signaling and regulatory pathways to manipulate the yeast behaviors in response to stimuli allows us to investigate every single salt determinant. The transformed yeasts were stressed with 2 M NaCl and 53 single genes responsible for salt tolerance in transgenic yeasts were rescued. Most of the isolated genes were found to be homologues of proteins known to be associated with stress tolerance in other plant systems, including late embryogenesis abundant proteins (LEAs), transporters, DNA/RNA-binding proteins, proteases, osmotic regulation proteins, photosynthesis-related proteins and enzymes related to metabolism (Table 2). Thirty-nine of the isolated total 53 genes recapture the salt tolerance in re-transformed yeasts (Figure 6), correlated to the expression profiles demonstrated by qRT-PCR after 400 mM salt treatment in *A. canescens* for those genes (Figure 5). Halophytes are famous for their higher salt-adaptive plasticity and special evolved salt glands, enabling elimination or sequestration of extra salt from metabolically active tissues [28]. In the presence of excess salt for glycophyte, the halophytes, such as *A. canescens* (like 400 mM NaCl in our condition), display optimum growth may be due to constitutive expression of genes that encode salt-tolerance determinants [29], or may be due to the adaptive and rapid regulation of gene expression in response to salt, which is consistent to our observations in this study. The qRT-PCR data revealed that the tested genes are responding to salt stress rapidly (within 6 h) and then probably are translated into the corresponding proteins, functioning to maintain the ion homeostasis, leading to the transcripts abundance reduction 24 h after salt stress application.

In this study, most of the isolated gene encoding proteins were enriched in plasma membrane associated category with protein binding activity after GO analysis (Figure 4). Among them, three genes encoding proteins with transporting activities were isolated: K^+^ uptake permease, hexose transporter and aquaporin NIP6.1 were isolated, which indicate the roles of transporter in plant adaption to salt stress. Transporters could facilitate the absorption of H_2_O and mineral nutrients to enable the maintenance of intracellular homeostasis [30,31,32]. Intriguingly, the largest group of proteins were enriched in photosynthesis, light harvesting in photosystem I in biological process in our GO analysis, indicative the roles of photosynthesis-related proteins in plant response to salt stress. Sugihara et al. characterized an oxygen evolving enhancer protein 1 from mangrove *Bruguiera gymnorrhiza* treated with 500 mM NaCl after two-dimensional gel electrophoresis and showed that the quantity of this protein increased after salt treatment [33]. One of our studies [34] demonstrated transgenic yeast cells expressing AcPsbQ1 (Ac469 in this study) exhibited better salt- and drought-stress survival rates. Transcriptomic and proteomic studies have shown that the expression levels of photosystem related proteins could be increased by abiotic stress at both transcriptional and translational level [9]. Under saline environments, *A. canescens* exhibits enhanced photosynthetic capacity in terms of increased net photosynthetic rate and water use efficiency [26], which was also reported in another halophyte *C. glauca* [6,8]. However, how the photosynthesis-related genes take use of the host yeast signaling pathways to play functions leading to enhanced yeast cell survival rates upon salt treatment is still unknown. Taken together, these studies indicate that photosystem proteins might play secondary roles in abiotic stress resistance in plants. However, the underlying functions and mechanisms of those proteins in plant abiotic stress resistance remains to be determined. Transcription factors (TFs), including an AP2-like ethylene-responsive TF (ERF), a zinc finger (C3HC4-type RING finger) family protein and an auxin-responsive factor (ARF) domain class TF, were isolated in our work. Regularly, TFs are considered to function as signaling regulator to manipulate transduction networks, there are emerging studies demonstrating that TFs are also responsible for stress tolerance when expressed in yeast [16,22,27]. A dehydration responsive element-binding factor (DREB) TF ZmDBF3 from maize (*Zea mays* L.) was demonstrated to increase host survival ability to salt, osmotic and extreme temperature stress when expressed in *E. coli*, yeast and *Arabidopsis* [35]. *EsDREB2B*, a DREB TF from *Eremosparton songoricum*, dramatically improved host yeast cells growth compared to the control yeast when stressed with Polyethylene glycol (PEG), NaCl, and heat [36]. Four protein members (ScDREB3, ScDREB5, ScDREB8, and ScDREB10) from the A-5 subgroup of the DREB subfamily in a typical desert moss, *Syntrichia caninervis*, enhanced the yeast's salt, drought, or cold tolerance [37]. These well-demonstrated examples revealed that TFs may function in yeast expression system via regulating conserved signaling pathway. Late embryogenesis abundant (LEA) proteins have long been associated with abiotic stress in plants. In our screen, two different LEA proteins were isolated and the transformed yeast cells recaptured salt stress tolerance, of which the transcription level were found to be positively regulated by salt stress and low temperature (Figure 5 and Figure S3 in the Supplementary Material). One LEA gene (Ac1723) is the only gene isolated in this study that is responsive to salt, drought and cold stress treatment. LEA proteins are typically stress inducible and several studies have confirmed LEA proteins act as water-binding molecules that function in ion sequestration, in macromolecule membrane stabilization and in preventing molecular denaturation [38,39].

In this study, we employed yeast expression system to isolate salt tolerance-related genes from halophyte *A. canescens* and the separated genes are capable to enhance the survival capacity in transformed yeast cells and the gene expression are responsive to salt stress treatment. However, yeast-specific glycosylation and other post-translational modifications may be different from modifications found in plant systems [40]. Our further work will focus on characterization of the functionalities of these isolated genes in the context of plant expression system, in order to more fully characterize salt resistance in halophytes.

## 4. Materials and Methods

### 4.1. Plant Materials, Plasmid, Yeast and Escherichia coli Strains, Medium

Xero-halophyte *A. canescens* was used in this study and the cDNA library generation was described in our previous report [20]. Briefly, surface-sterilized seeds were germinated and the seedlings were transferred to 1/2 Hoagland (pH 6.0) solution in controlled environmental facility. After 50 days of growth, plants were then subjected to 400 mM NaCl treatment for 48 h, and the harvested samples (young leaves and stems) were immediately frozen with liquid nitrogen and kept at −80 °C for RNA preparation and cDNA library construction. The gateway system compatible vector pDONR222 was used for entry cloning and pYES-DEST52 [20] was used for functional expression in yeast. The *E. coli* strain DB3.1 was used to maintain the gateway compatible backbones pDONR222 and pYES-DEST52 with a *ccdB* cassette. And *E. coli* DH10B competent cells were used to propagate and maintain both pDONR222 harboring cDNA library and pYES-DEST52-genes library. The yeast *S. cerevisiae* strain INVSc1 (*MATa his3D1 leu2 trp1-289 ura3-52 MAThis3D1 leu2 trp1-289 ura3-52*) was employed in this study (Invitrogen, Shanghai, China). Yeast cells were grown in either YPDA medium (with 2% glucose, 2% peptone, 1% yeast extract and 2% agar) or on an uracil-deficient synthetic complete (SC-U) medium (Fungenome Co., Beijing, China) with 2% (*w/v*) glucose for normal growth or 2% (*w/v*) galactose during induction and selection at 28 °C. 

### 4.2. Yeast Functional Screening to Select the Salinity Tolerant Transformants

To express the cDNA library in yeast, a plasmid mixture harboring cDNA library was introduced into 10 vials of 100 μL INVSc1 competent cells using a PEG-lithium acetate method and the resultant transformed yeast was streaked on 2% glucose SC-U plate and incubated for 48–72 h at 28 °C. After yeast colony was visualized, more than 1 × 10^5^ transformants were picked up randomly and inoculated into 500 μL SC-U (+2% galactose) in 1.5 mL Eppendorf individually with a 200 rpm shaking at 28 °C. After 24 h inoculation, 2 μL transformed yeast was spotted on SC-U plates (2% agar + 2% galactose) with 2 M NaCl and then incubated at 28 °C for 48–72 h and photographed.

Before screening of salt-related transformants, an optimization of salt selection concentration was performed. Generally, INVSc1 with pYES-DEST52 and INVSc1 alone were streaked on SC-U or SC agar plates plus 2% galactose and various NaCl concentrations (1 M, 2 M and 3 M) and then grown at 28 °C for colony visualization.

### 4.3. Plasmid Rescue, Sequencing and Data Analysis

Isolated yeast transformants were grown each in 10 mL SC-U medium containing 2% glucose for 24 h with a 200 rpm shaking at 28 °C. The pellets were then subjected to 50 U/μL lyticase (Sigma-Aldrich, St. Louis, MO, USA) and 1 M sorbitol to generate protoplast, and then lysed with 200 mM NaOH and 10 g/L SDS. Nucleic acid fraction recovered from yeast were purified with two sequential rounds of phenol: chloroform: isopropanol (25:24:1) extraction, followed by isopropyl alcohol precipitation of nucleic acids. To analyze the inserts cloned into pYES-DEST52 based yeast expression plasmid, nucleic acid preparations recovered from individual yeast transformants showing resistance to salinity stress were back transformed into *E. coli* DH5α via electroporation (Gene Pulser Xcell Electroporation System, BioRad, Hercules, CA, USA). *E. coli* back transformants were analyzed for the presence of inserts by colony PCR with primer pair T7 and pYES-R (Table S1) on the backbone pYES-DEST52. *E. coli* re-transformants were then subjected to plasmid-prep and sequencing. Sequencing of *Atriplex* cDNA conferring salinity tolerance was performed at Beijing Genomic Institute, China, with primer pair T7 and pYES-R (Table S1). After sequencing, vector sequences were trimmed using SeqMan II (DNASTAR, Inc., Madison, WI, USA) and NCBI VecScreen (http://www.ncbi.nlm.nih.gov/tools/vecscreen/). Trimmed cDNA sequences were assembled using Vector NTI (Invitrogen, Carlsbad, CA, USA). Contigs were built using the CAP3 assembly program [41] with the parameters set at 95% identity over 40 bp. Individual processed unique genes were proceeded to Basic Local Alignment Search Tool (BLASTX) analysis against the non-redundant (nr) database (http://www.ncbi.nlm.nih.gov) with a cut-off *E*-value ≤ 10^−5^. All consequential *Atriplex* sequences were then deposited in GenBank database and were subjected to data analyses.

### 4.4. Functional Annotation and Categorization

The complete sequence of the *A. canescens* genome is not yet available, whereas that of model dicotyledon plant *A. thaliana* is available, and its bioinformatics tools are available online. All the *Atriplex* cDNA sequences isolated were matched to *A. thaliana* transcripts manually (https://www.arabidopsis.org/). For each encoded predicted peptide, the most similar one with lowest *E*-value was considered to be the best match. Gene ontology (GO) information were extracted from GO annotation file downloaded from the TAIR website online (http://www.arabidopsis.org/). GO enrichment was conducted to identify the related biological modules. Fisher’s exact test was used and *p* < 0.05 was considered to indicate a statistically significant difference to identify the three principles of GO, including “Biological Process” (BP), “Cellular Component” (CC) and “Molecular Function” (MF).

### 4.5. Quantitative RT-PCR Validation of Salt-Related Genes

The expression profiles after salt treatment of isolated salinity resistance candidate genes were investigated by performing qRT-PCR. Two month-old well-grown and healthy *A. canescens* cultured in Hoagland solution were complemented with different abiotic stressors (400 mM NaCl, 20% PEG6000 (*w/v*) or 4 °C) and nontreated control, respectively. Young leaves and stems, the materials of which we used for cDNA library construction, were sampled at 0, 6, 12, 24 and 48 h after stress treatments, and were immediately snap-frozen in liquid nitrogen and stored at −80 °C for use. RNA preparation and qRT-PCR analysis was performed according to our previous description [25]. qRT-PCR was repeated three times with different batches of treated plants and one of the representative data was shown. Gene expression values are normalized relative to the internal control *AcEF1α*. Values are the means ± standard deviation (SD) (*n* = 3). The probe sets for real-time PCR and the corresponding primer pairs used for the analysis are listed in Table S1. 

### 4.6. Salt-Stress Assay for Isolated Salt Resistance Genes

The candidate genes were retransformed into yeast INVSc1 cells and challenged with high concentrations of NaCl to evaluate their performance. Yeast cells containing pYES EV (control) or pYES52- *Acgenes* were grown in SC-U medium with shaking (200 rpm) at 28 °C for 24 h and their OD_600_ were diluted with SC-U to five different levels: 1, 10^−1^, 10^−2^, 10^−3^, 10^−4^ gradually. Two µL of each culture was spotted onto SC-U plates plus 2 M NaCl. The plates were incubated at 28 °C for 4 days and then photographed.

## Figures and Tables

**Table 1:** General information *A. canescens* full-length cDNA library. Recombinant rate represents the percentage of cDNA inserts in plasmid checked by PCR and electrophoresis.

cDNA library	Vector	CFU	Average Inserted Fragment	Recombinant Rate
Primary cDNA library	pDONR222	1.76 × 10^6^	>1 kb	91%
Destination cDNA library	pYES-DEST52	1.624 × 10^7^	>1 kb	95%

**Table 2:** Annotation of 53 isolated genes from *A. canescens* exploiting yeast functional screening system.

Clone No.	GenBank Accession No.	Putative Gene Function	Organism Matched	Full-Length ORF	*E*-Value	Target GenBank Accession No.	Recapturing Salt Resistance
Ac113	KJ026988	Cysteine desulfurase	*Arabidopsis thaliana*	Yes	2 × 10^−70^	NP_001078802.1	No
Ac151	KJ026991	Phosphoglyceride transfer family protein	*Theobroma cacao*	Yes	5 × 10^−160^	EOY17339.1	No
Ac152	KJ026992	Cyclophilin	*Suaeda salsa*	Yes	5 × 10^−92^	AGI78541.1	Yes
Ac264	KJ026993	Phosphorylase superfamily protein	*Theobroma cacao*	Yes	5 × 10^−40^	EOY10097.1	No
Ac273	KJ026995	Beta-amylase	*Ricinus communis*	Yes	3 × 10^−21^	XP_002519919.1	No
Ac313	KJ026998	Membrane-associated progesterone binding protein	*Theobroma cacao*	Yes	4 × 10^−46^	EOY29287.1	Yes
Ac315	KJ027000	Signal recognition particle receptor	*Theobroma cacao*	Yes	7 × 10^−122^	EOY25233.1	Yes
Ac323	KJ027001	Pre-mRNA cleavage complex II protein Clp1	*Zea mays*	No	9 × 10^−113^	AFW66051.1	Yes
Ac353	KJ027002	K^+^ uptake permease 7	*Theobroma cacao*	No	2 × 10^−119^	EOX98796.1	Yes
Ac354	KJ027003	Chlorophyll a/b-binding protein	*Solanum lycopersicum*	Yes	8 × 10^−144^	AAA34140.1	No
Ac359	KJ027004	Cytochrome c1-1	*Solanum lycopersicum*	Yes	2 × 10^−174^	XP_004231797.1	No
Ac375	KJ027006	Glycogen synthase kinase-3	*Ricinus communis*	No	2 × 10^−149^	XP_002522231.1	Yes
Ac392	KJ027007	Major chlorophyll a/b binding protein LHCb1.2	*Spinacia oleracea*	Yes	0	CAJ77390.1	No
Ac435	KJ027013	3-ketoacyl-CoA thiolase 2, peroxisomal-like	*Solanum lycopersicum*	Yes	0	XP_004247828.1	Yes
Ac438	KJ027014	Glycine-rich protein	*Pisum sativum*	Yes	6 × 10^−3^	CAH40798.1	Yes
Ac441	KJ027018	Zinc metalloprotease	*Ricinus communis*	No	4 × 10^−63^	XP_002518787.1	Yes
Ac454	KJ027022	RNA-binding family protein with retrovirus zinc finger-like domain	*Theobroma cacao*	Yes	2 × 10^−66^	EOY18432.1	Yes
Ac458	KJ027023	Cytochrome P450	*Populus trichocarpa*	Yes	1 × 10^−104^	XP_002334834.1	Yes
Ac469	KJ027025	Oxygen-evolving enhancer protein 3	*Spinacia oleracea*	Yes	8 × 10^−130^	P12301.1	Yes
Ac529	KJ027035	Temperature-induced lipocalin	*Populus balsamifera*	Yes	2 × 10^−80^	ABB02389.1	Yes
Ac549	KJ027036	Late embryogenesis abundant protein	*Ricinus communis*	Yes	5 × 10^−07^	XP_002509455.1	Yes
Ac567	KJ027038	Polyubiquitin family protein	*Populus trichocarpa*	No	0	XP_006372519.1	No
Ac574	KJ027042	Tetratricopeptide repeat (TPR)-like superfamily protein	*Theobroma cacao*	Yes	2 × 10^−104^	EOY31556.1	Yes
Ac590	KJ027045	Ubiquitin	*Arabidopsis thaliana*	No	4 × 10^−55^	ABH08753.1	Yes
Ac592	KJ027046	Zinc finger (C3HC4-type RING finger) family protein	*Arabidopsis thaliana*	Yes	1 × 10^−94^	NP_564060.1	Yes
Ac601	KJ027047	ARF domain class transcription factor	*Malus domestica*	No	4 × 10^−88^	ADL36578.1	Yes
Ac603	KJ027048	Dehydration-responsive element binding protein	*Atriplex canescens*	Yes	0	AEW68339.1	No
Ac602	KJ027049	Cysteine proteinase A494	*Populus trichocarpa*	Yes	0	XP_002305451.2	Yes
Ac622	KJ027051	AP2-like ethylene-responsive transcription factor	*Medicago truncatula*	No	1 × 10^−63^	XP_003625138.1	Yes
Ac799	KJ027055	S-adenosyl-L-homocysteine hydrolase	*Beta vulgaris*	Yes	0	BAE07182.1	No
Ac809	KJ027057	Alanine aminotransferase 2	*Theobroma cacao*	Yes	3 × 10^−113^	EOY27390.1	Yes
Ac812	KJ027058	Armadillo/beta-catenin repeat family protein	*Theobroma cacao*	Yes	3 × 10^−115^	EOY18778.1	Yes
Ac824	KJ027061	Hexose transporter	*Elaeis guineensis*	Yes	8 × 10^−123^	AEQ94177.1	Yes
Ac1455	KJ027067	Zinc finger and BTB domain-containing protein	*Theobroma cacao*	No	2 × 10^−61^	EOY17025.1	Yes
Ac1458	KJ027069	Thiamin biosynthetic enzyme	*Glycine max*	Yes	0	BAA88226.1	No
Ac1463	KJ027070	GTP-binding protein sar1	*Ricinus communis*	No	1 × 10^−121^	XP_002515297.1	Yes
Ac1476	KJ027073	Nonspecific lipid-transfer protein-like protein At2g13820-like	*Vitis vinifera*	No	9 × 10^−35^	XP_003632312.1	Yes
Ac1502	KJ027076	NAD(P)H dehydrogenase C1 isoform 2	*Theobroma cacao*	No	7 × 10^−153^	EOX92707.1	Yes
Ac1607	KJ027083	Profilin	*Chenopodium album*	No	6 × 10^−83^	Q84V37.1	Yes
Ac1614	KJ027084	Leucine-rich repeat protein kinase family protein	*Arabidopsis thaliana*	No	3 × 10^−27^	NP_190219.1	Yes
Ac1625	KJ027085	eukaryotic elongation factor 1A	*Salsola komarovii*	No	2 × 10^−160^	BAC22127.1	Yes
Ac1637	KJ027087	Ferredoxin-1	*Mesembryanthemum crystallinum*	No	4 × 10^−66^	O04683.1	No
Ac1647	KJ027088	RNA-binding family protein	*Theobroma cacao*	Yes	4 × 10^−71^	EOY04889.1	Yes
Ac1723	KJ027090	Late embryogenesis abundant protein D-113	*Gossypium hirsutum*	No	1 × 10^−6^	P09441.2	Yes
Ac1737	KJ027095	Glyceraldehyde-3-phosphate dehydrogenase	*Atriplex nummularia*	No	0	P34783.1	No
Ac1752	KJ027097	Putative ankyrin-repeat protein	*Vitis aestivalis*	No	4 × 10^−104^	AAQ96339.1	No
Ac1774	KJ027102	Cysteine proteinases	*Theobroma cacao*	Yes	4 × 10^−115^	EOX95504.1	Yes
Ac1786	KJ027105	Calcium-dependent lipid-binding (CaLB domain) plant phosphoribosyltransferase	*Theobroma cacao*	Yes	0	EOY09444.1	Yes
Ac1861	KJ027110	calmodulin1	*Zea mays*	Yes	5 × 10^−98^	AFW78488.1	Yes
Ac1883	KJ027112	Abscisic acid stress ripening protein	*Mesembryanthemum crystallinum*	Yes	9 × 10^−^^5^	AAC14177.1	Yes
Ac1913	KJ027114	Non-specific lipid-transfer protein	*Beta vulgaris*	Yes	2 × 10^−24^	Q43748.1	Yes
Ac2594	KJ027115	Aquaporin NIP6.1 family protein	*Populus trichocarpa*	Yes	6 × 10^−135^	XP_002304723.1	Yes
Ac2611	KJ027117	23 kDa precursor protein of the oxygen-evolving complex	*Salicornia europaea*	Yes	4 × 10^−155^	BAG70022.1	Yes

## Supplementary Materials

Supplementary materials can be found at www.mdpi.com/1422-0067/18/11/2444/s1.
