# Supplementary material for: Exploration for the Salinity Tolerance-Related Genes from Xero-Halophyte Atriplex canescens Exploiting Yeast Functional Screening System
Source: Int J Mol Sci. 2017 Nov 17;18(11):2444. doi: 10.3390/ijms18112444 (PMC5713411; doi:10.3390/ijms18112444)
Supplement: Supplementary file 1 [file ijms-18-02444-s001.pdf]

Supplementary figure legend

Figure S1. Quantitative real-time PCR validation of selected genes under salt-stress treatment. Hydroponically grown *A. canescens* was treated with 400 mM NaCl and sampled at different time points, and the isolated RNA was subjected to cDNA synthesis and qRT-PCR. qRT-PCR was repeated three times with different batches of treated plants and one of the representative data was shown. Gene expression values are normalized relative to *AcEF1α*. Values are the means ± standard deviation (SD) (n = 3).

Figure S2. Quantitative real-time PCR validation of selected genes under drought-stress treatment. Hydroponically grown *A. canescens* was treated with 20% PEG6000 and sampled at different time points, and the isolated RNA was subjected to cDNA synthesis and qRT-PCR. qRT-PCR was repeated three times with different batches of treated plants and one of the representative data was shown. Gene expression values are normalized relative to *AcEF1α*. Values are the means ± standard deviation (SD) (n = 3).

Figure S3. Quantitative real-time PCR validation of selected genes under low temperature stress treatment. Hydroponically grown *A. canescens* was placed at 4℃ and sampled at different time points, and the isolated RNA was subjected to cDNA synthesis and qRT-PCR. qRT-PCR was repeated three times with different batches of treated plants and one of the representative data was shown. Gene expression values are normalized relative to *AcEF1α*. Values are the means ± standard deviation (SD) (n = 3).

Figure S4. Venn diagram of transcripts profiling of salt resistance related genes under salt, drought, and low temperature treatment. Sixteen out of 28 salt stress responsive genes exhibited induction by PEG6000 treatment, while only 6 of 28 salt stress responsive genes showed transcript accumulation changes after low temperature treatment. Only 1 of 28 genes were transcriptionally induced by salt, drought and low temperature.

Figure S1.

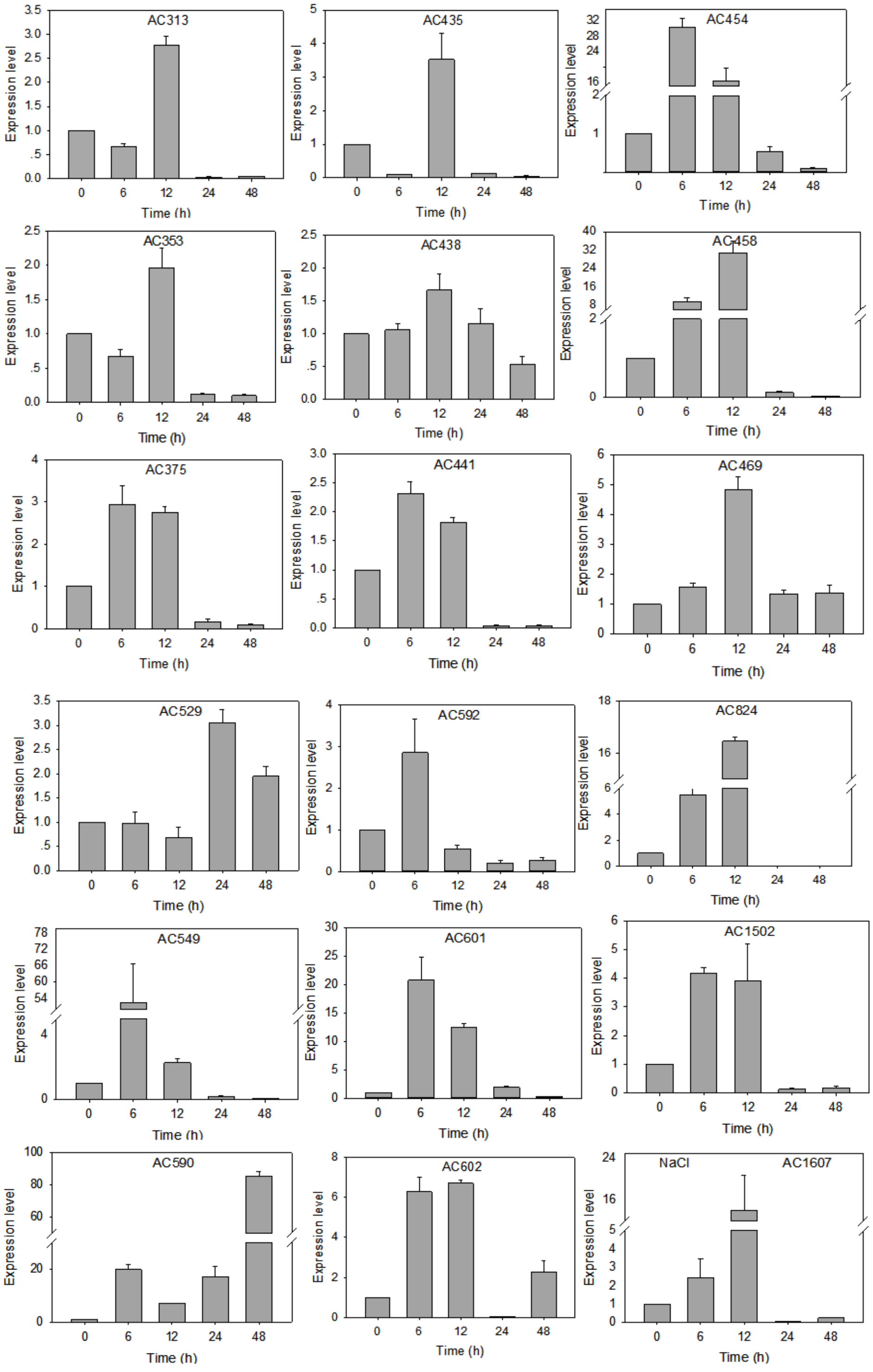
AC313
AC435
AC454
AC353
AC438
AC458
AC375
AC441
AC469
AC529
AC592
AC824
AC549
AC601
AC1502
AC590
AC602
NaCl
AC1607
Expression level
Time (h)
0
6
12
24
48

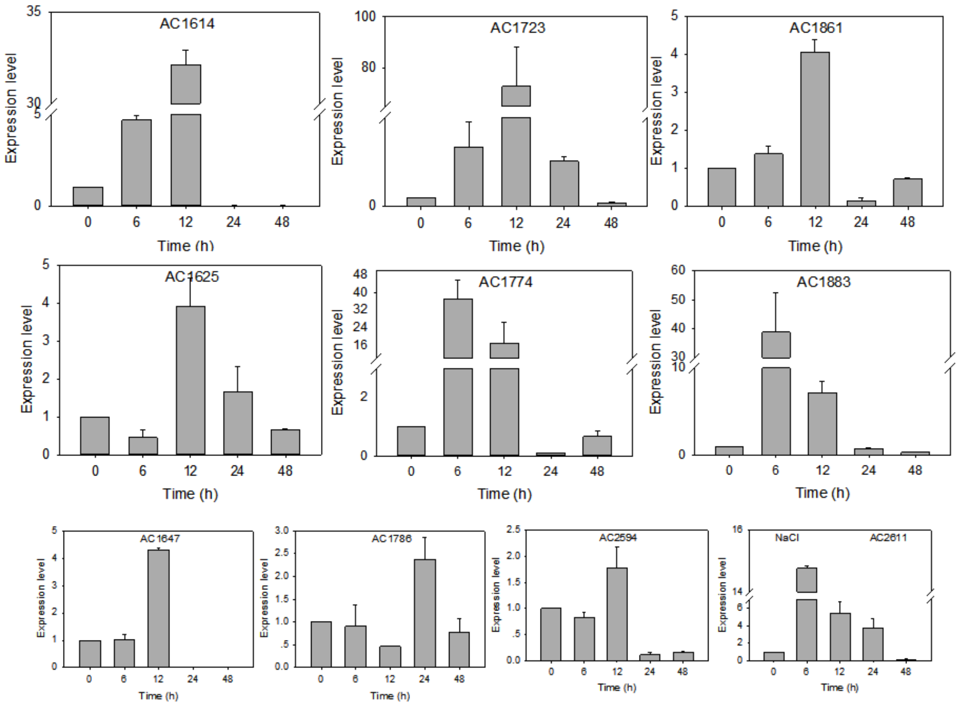
AC1614
AC1723
AC1861
AC1625
AC1774
AC1883
AC1647
AC1786
AC2594
NaCl
AC2611
Expression level
Time (h)

Figure S2.

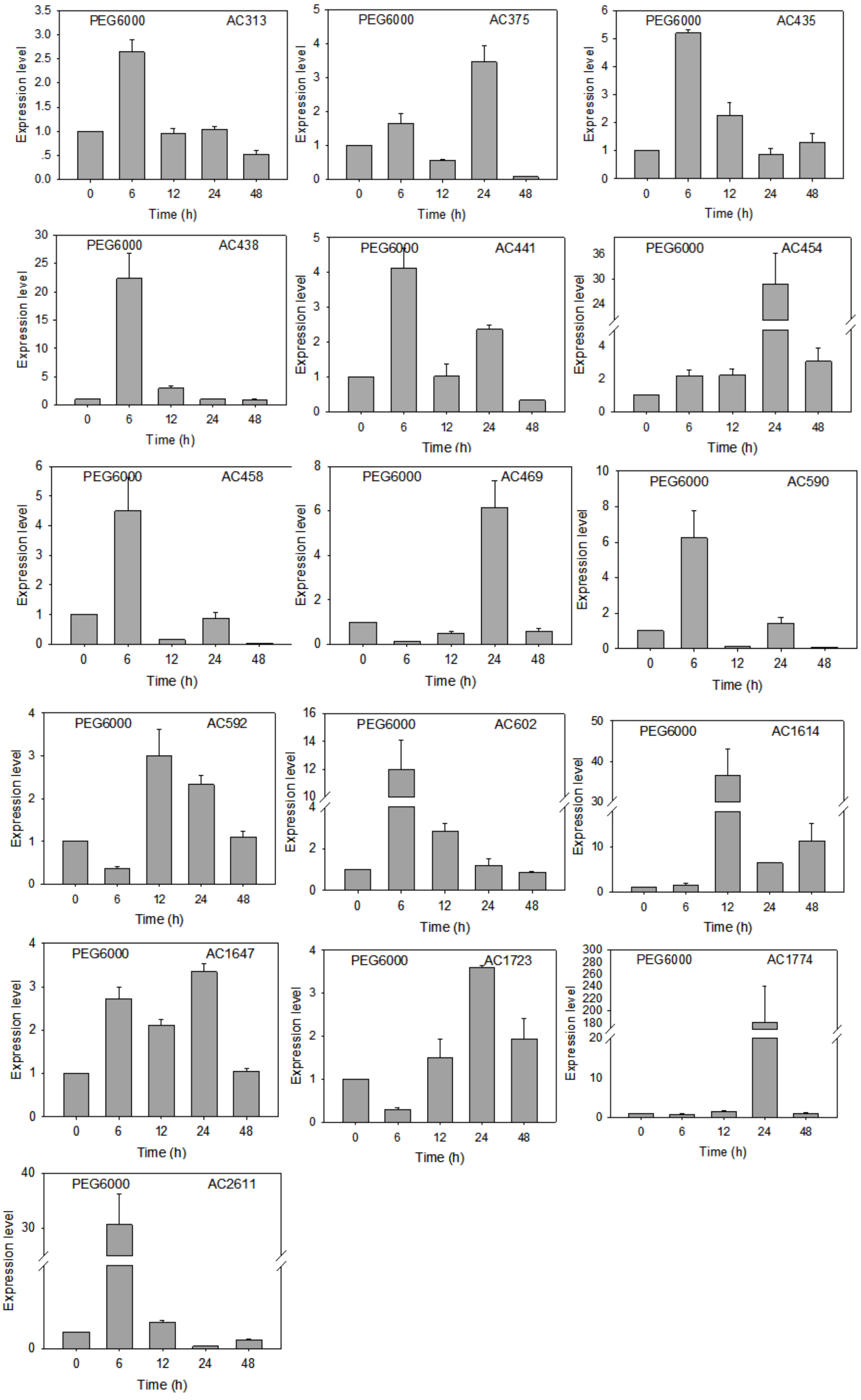

PEG6000
AC313
AC375
AC435
AC438
AC441
AC454
AC458
AC469
AC590
AC592
AC602
AC1614
AC1647
AC1723
AC1774
AC2611
Expression level
Time (h)

Figure S3.

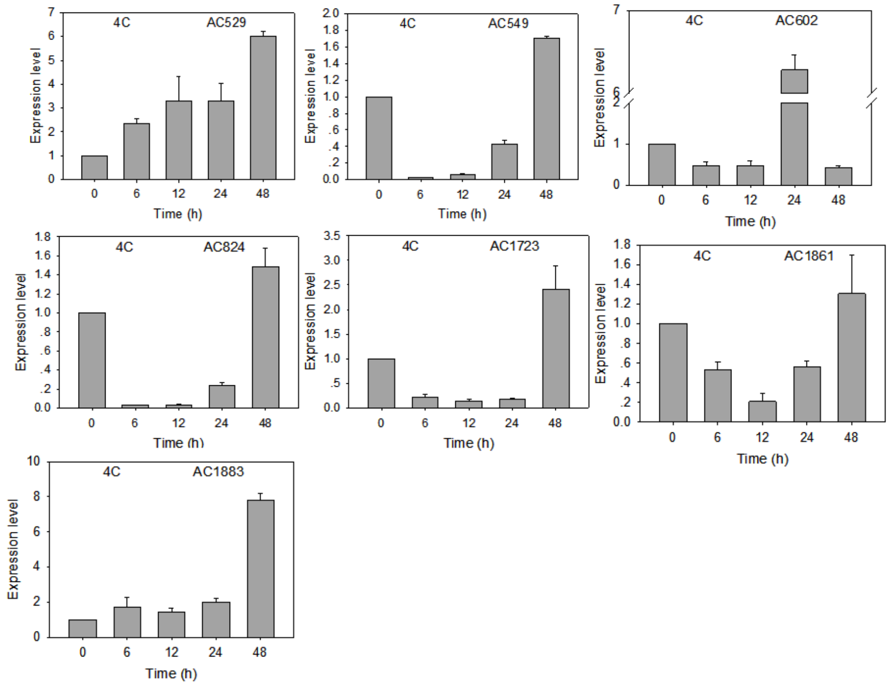

4C
AC529
Expression level
Time (h)
4C
AC549
Expression level
Time (h)
4C
AC602
Expression level
Time (h)
4C
AC824
Expression level
Time (h)
4C
AC1723
Expression level
Time (h)
4C
AC1861
Expression level
Time (h)
4C
AC1883
Expression level
Time (h)

Figure S4.

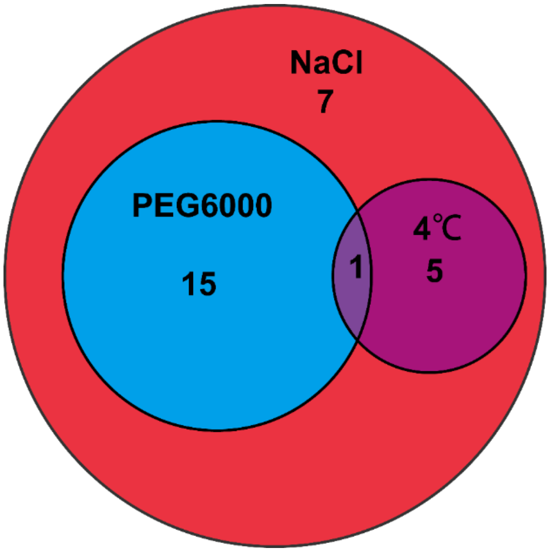

Supplementary Table 1. List of primers used in this study.

Primer name	Primer sequence
EF1α-F	CCCCAGTTCTCGACTGTCAC
EF1α-R	TGGTGGGAACCATCTTCACG
T7	TAATACGACTCACTATAGGG
pYES-R	AGGGTTAGGGATAGGCTTACCTTC
113-F	TAGGTTCGGGATTGGGAGG
113-R	AGAGGGCTCATCTCCCTCAA
149-F	AGGCATTGCCCGATGAACTT
149-R	TGTCGCAGCATTGGACTAGG
152-F	ATGTGCCAAGGTGGGGATTT
152-R	CCGGTCCTGTATGCTTCAACT
264-F	AACTACGGTGAACGCCCTAC
264-R	GGCCTTTCCATAAGCCTCG
273-F	GGTCTCCGGCAATACGGTAA
273-R	ATCAGGCTCAACCATCCAGC
313-F	GGGTTTTCGACGTTTCTGCC
313-R	GCTAGAGCTCTGCTAGCGTC
323-F	TTGCCAAGGATCCGGAAGAC
323-R	CCTGCTGATGGTGCAAGGTA
353-F	AGGGGTGAGGCTAGACACTT
353-R	AACAACCCCGGGATTCCTTG
375-F	CGTCCATTGCCACCTCTCTT
375-R	TACTGTCGCCTCACATGCTC
392-F	CGGTGGTAGCTTTGACCCAT
392-R	GGCCCTTTCCGGTGACAATA
433-F	AATGGGCAATCTGCGTGTGA
433-R	GTCACCATTGCGCCATCCTA
435-F	GATGAAGCGCCGTGGTAAAG
435-R	ACGAGCGTTGCTTAGGTCAT
438-F	ATCACAGTCCGTGGAGGAGT
438-R	GTCCACCACCATAGTGACCC
441-F	CGACACTTGTTGGGAATCACAG
441-R	GCCACTGCAACCACAATTCC
444-F	CGATCACTTACGGCGGAACA
444-R	ATTCCAAGCCACCGCCTATT

454-F	TCTAGGGTTTTCGTGCTCCG
454-R	ACAAGCGGCCAACATACAGA
458-F	AGCCAAAGGATGACTTGCTGA
458-R	TTGAAGTTGTGCCTTGGCCT
521-F	TGCCGGTGTCAGGTTTACTC
521-R<br>529-F	AGCTCATCGGCATAGGAAGC<br>TTTCAGCCAAAAGACGGGGA
529-R	ATCGGAATTTGGGTCGGCTT
549-1-F	ACATGCTGGAGGAGACGAAG
549-1-R	TTAACATCGGCAGCGGTCTT
549-2-F	TGAAGTCTCACCAACGAGCC
549-2-R	AGCGGTCTTAGTAGTCGGGT
567-F	CGTTCCCGAAATCAACGTCAAA
567-R	ACCTCGAGAGTTATGGTTTTGC
574-F	TGAACCTGACACGACTTCCG
574-R	GTTGTTCGTCGTCGCCATTG
590-F	AGAGACTATGACCAGGCGGT
590-R	GTCCTCCCATCCTCCAGACT
592-F	GATGGATGCTGTGCTCCCTT
592-R	AGTGTTACCATGCCCATCCG
601-F	AAGGGCATGATTCCGATGCT
601-R	GGCTGCAGGCCTTGATGATA
602-F	GCAACAGTTGGTGGACTGTG
602-R	ACGTTCAATACCACCGGCTT
622-F	GCCTGCTGAGGGTGACTATC
622-R	TTTTACTCTGGCCCCACCAC
809-F	GTGGCCTGCTGAATGAGACT
809-R	TAAACGCTCAACAACGGCTG
812-F	ACTCTTCGAATGGTGGCTGG
812-R	CCCCTAGCTACCTGTGCAAC
824-2-F	TCTTCTTGCTCCCCGAAACT
824-2-R	GCATTAGCAGGCTTGATCGC
1455-F	TGCCAAGCTCAAAGTCACCA
1455-R	CTGTAACGGAGTTGGGGGTC
1463-F	GATGAGCTGCGCTATCACCT
1463-R	CCTTCCCCGTAGCCCATTTT
1476-F	AGCAAGCTAGGCGATGTTGT
1476-R	TGGACCTTGCAAGCAGTAGG
1502-F	TAAGCCTTGCCTTCCTCAGC
1502-R	CGTGGGTTACCCTTTACCCG
1566-F	GCTAAGGAGCGGGTGATGAA
1566-R	CAAGCGTGCAAAAAGCTTGC
1607-F	ATGTGCGACATCGACGGTAA
1607-R	ATGGCGCTTACCTCTTCAGG
1614-F	GCCGTCAAGGTGCTCTCTAA

1614-F	GCTGGGTCCTTCTTCACAGT
1625-F	CAGACAAGCCACTCCGTCTT
1625-R	GGGACCGAAGGTAACAAGCA
1637-F	ACCTGAGGGTACCCAGGAGA
1637-R	AACCTTACCGACGCATGTGG
1647-F	CACCTCAGGTTAATGATGCGG
1647-R	GGCACACTTCCCAACGTAGT
1723-F	GTGAGAAATGGATGCGGCGA
1723-R	TGATCCACCTTCTCCGTCAC
1726-F	GGAAATGTTGCGCTCTCAGT
1726-R	TGGGCTTGGGCTAAATGAGT
1752-F	GTTCTGTGCCAGACGAGTCA
1752-R	CCCTCCACATCACCAATGCT
1774-F	ATCTATGCCTGGCGCCATTT
1774-R	TGGAGTAGGGTATTCGGGCT
1786-F	GCCACCGCGTTGTTTGTTAT
1786-R	ACCTTGGGTGTCTTAGCACG
1853-F	AGAGGGCTCGTCTTGGACTA
1853-R	CCATTTGGCATGAGCAGAACA
1861-F	CAAGGAGCTCGGAACAGTGA
1861-R	CCATTTCCATCAGCATCGACC
1883-F	ATACACCGAGACCACCACCA
1883-R	AGCTCACCCAAATGCTCCTT
1912-F	TCACTTTCACCCACCCCTCT
1912-R	TGGCCGCTTTCTCCCAAATA
2594-F	AGAGACGCTTATCGGCCTTG
2594-R	ATGGTGATGGACGGGTTGAG
2611-F	GGTTGGCTCTCACTGTCCTC
2611-R	AATCCTTCGCCGTTGTAGGG
2623-F	CGAGCAAGTACCCCTCACAC
2623-R	GTTGAGCCAGAGGTGCTACA
